# Impacting factors and sources of perceived stress by home-quarantined residents in Shanghai during COVID-19 epidemic

**DOI:** 10.1186/s12889-023-15701-z

**Published:** 2023-04-28

**Authors:** Yiwei Zhou, Zhihui Chen, Wancang Li, Siwei Chen, Haiyun Xu, Zumu Zhou

**Affiliations:** 1 Business School, University of Shanghai for Science and Technology, Shanghai, China; 2 Department of Infection Control, Wenzhou People’s Hospital, Wenzhou, China; 3 Department of Health Assessment, Wenzhou Center for Disease Control and Prevention, Wenzhou, China; 4 School of Mental Health, Wenzhou Medical University, Wenzhou, China; 5 The Affiliated Kangning Hospital of Wenzhou Medical University Zhejiang Provincial Clinical Research Center for Mental Disorders, Wenzhou, China

**Keywords:** COVID-19, Epidemic, Home-quarantine, Omicron variants, Stress

## Abstract

**Background:**

Home-quarantine is one of the most common measures implemented to prevent or minimize the transmission of COVID-19 among communities. This study assessed stress levels of the home-quarantined residents in Shanghai during a massive wave of COVID-19 epidemic this year, explored the stress sources perceived by the respondents, and analyzed the association between each of the sociodemographic factors and the stress level.

**Methods:**

This online survey was launched during April 23 - 30, 2022, the early stage of a massive wave of COVID-19 in Shanghai, China. Participants were quarantined-residents negative for COVID-19. They were asked to list some situations that were their major concerns and perceived stressful, in addition to sociodemographic and COVID-19 related information. Moreover, they were asked to complete the Perceived Stress Scale-14 (PSS-14) for the assessment of stress level.

**Results:**

A total of 488 valid questionnaires were collected from 192 male and 296 female respondents. Overall, 207 persons (42.42%) presented high stress level (PSS-14 score ≥43). The top three concerns perceived stressful by respondents are “not allowed to go outdoors”, “uncertain duration of the epidemic”, and “lack of food supply”. Fewer than 50% of the respondents perceived the other situations stressful. Higher proportions of young adults (≤ 29 years old), males, unemployed, singles, and those with low income (≤ 1999 yuan/month) perceived high stress compared to their counterparts, none of COVID-19 related factors is associated with the stress level, including location of residence, result of nucleic acid test, knowledge about COVID-19, whether vaccinated, and quarantine duration.

**Conclusion:**

Home-quarantine applied to people negative for COVID-19 led to a lot of major concerns that may be perceived stressful, whereas the virus-related factors did not show significant impact on mental health of the respondents.

## Background

COVID-19 is a contagious disease caused by a Coronavirus identified in December 2019. The first known cases identified in Wuhan , China, showed severe acute respiratory syndrome thus named as SARS-CoV-2 [1]. The disease quickly spread worldwide and resulted in the COVID-19 pandemic. On January 30, 2020, WHO declared that the COVID-19 pandemic constituted a public health emergency of international concern [2, 3]. As of 20 April, 2022, there had been 50.4 million confirmed cases of COVID-19, including 6.2 million deaths directly attributable to COVID-19 [4].

During the last nearly three years, COVID-19 has been buffeting almost all the nations on the planet, and numerous variants of the virus have been reported, each with different characteristics. For example, Delta variant was characterized with increased transmissibility, severe disease course, and reduced effectiveness of treatments [5]. Omicron variant is a new heavily mutated COVID-19 variant with enhanced transmissibility and partial resistance to immunity induced by COVID-19 vaccines [6]. However, infections with the Omicron variant were reported to have milder severity in South Africa [7] and elsewhere [8, 9].

China has long held a “zero COVID” policy which advocates for large scale viral nucleic acid and antigen screening, quarantine of infected cases and close contacts in shelter hospitals and hotels, respectively, and lockdown of districts with severe outbreak [10]. This policy had been effective in preventing COVID-19 from overwhelming the hospital system until March 2022, when a massive wave of COVID-19 infected people in cities ranging from Shenzhen to Qingdao and Shanghai. Of these COVID-19 epidemics, the most severe one is that happened in Shanghai as evidenced by the most infected people and the longest duration of the epidemic. As of May 4, 2022, 593 336 cases had been identified, including 538 450 asymptomatic carriers. 503 people had died with or from COVID-19, according to the Shanghai Municipal Health Commission. The virus strain was identified as Omicron BA.2 and BA.2.2 variants [10].

On March 28, Shanghai municipal government started to implement the so-called “static administration” in some districts of the city, including Pudong, Puxi, Punan and adjacent areas in accordance with the “zero-COVID-19” policy. In addition to large-scale viral nucleic acid and antigen screening, quarantine of infected cases and close contacts in shelter hospitals and hotels, this policy requires 1) all public officials to work at home, except for the personnel involving in the epidemic prevention work; 2) except for the key enterprises designated by the city and the public service enterprises that protect people's livelihood, all shopping malls, hotels, restaurants and entertainment establishments and other business premises must be suspended; 3) no public buses, taxis and online ride-hailing services are allowed to run; 4) residents are not allowed to go outdoors and gather. Although these restrictions are effective in minimizing epidemic of COVID-19, they may have more extensive impact on the mental health of people, in addition to restricting people’s daily life and damaging economic system.

This study assessed stress levels of the home-quarantined residents in some districts of Shanghai during the early stage of a massive wave of COVID-19 epidemic which started in early March and decayed in June 2022. We explored the stress sources perceived by the home-quarantined people, analyzed the association between each of the sociodemographic factors and stress level perceived by the respondents, and found out risk factors that increased stress level in the respondents.

## Materials and methods

### Participants

This cross-sectional survey was carried out online by using an online questionnaire through the Wenjuanxing platform (Survey Star Platform, Changsha Ranxing Science and Technology, Shanghai, China) between April 23 and 30, 2022, the early stage of COVID-19 epidemic in Shanghai. A qualified participant must be ≥ 13 years old and was living in the forementioned districts of Shanghai as a resident rather than a traveler. Participants were asked to provide sociodemographic information including gender, age, education level, marital status, occupation, employment status, location of residence, plus the information directly related to COVID-19 epidemic such as nucleic acid test results, vaccinated or not, knowledge about COVID-19, and duration of home-quarantine (see Table 1). All the information provided by participants was kept confidential and would not be used for any other purpose as stated in the study protocol, which was reviewed and approved by the Institutional Review Board of the Affiliated Kangning Hospital of Wenzhou Medical University, China.

**Table 1:** Descriptive characteristics of the participants (*N* = 488)

Characteristics	n	%
**Gender**		
Male	192	39.34
Female	296	60.66
**Age**		
≤29	221	45.29
30-49	187	38.32
≥50	80	16.39
**Employment status**		
Unemployment	184	37.71
Employment	263	53.89
Retired	41	8.40
**Education level**		
High school or below	83	17.01
Undergraduate	211	43.24
Postgraduate	194	39.75
**Occupation**		
Engineer or technique	52	10.66
Business or IT	98	20.08
Medicine or education	209	42.83
Administer	26	5.33
Agriculture or forestry	103	21.10
**Income/month (RMB)**		
≤1,999	157	32.17
2,000-4,999	84	17.21
5,000-9,999	103	21.11
≥10,000	144	29.51
**Marital status**		
Married	244	50.00
Single	244	50.00
**Location of residence**		
High risk area	243	49.80
Medium risk area	142	29.10
Low risk area	103	21.11
**Nucleic acid test**		
Positive	14	2.87
Negative	474	97.13
**Knowledge about COVID-19**		
No or little	274	56.14
Some	172	35.25
Very knowledgeable	42	8.61
**Vaccinated**		
Yes	459	95.43
Not yet	29	4.57
**Quarantined for (days)**		
≤9	20	4.10
10-19	49	10.04
20-29	201	41.19
≥30	218	44.67

### Stress sources and assessment

In addition to providing sociodemographic information and those directly related to COVID-19 epidemic as mentioned before, respondents were asked to list some situations that were their major concerns and perceived stressful during the home-quarantine duration. Moreover, participants were asked to complete the Perceived Stress Scale-14 (PSS-14), which is a widely used psychological instrument for measuring the perception of stress. The original PSS-14 was compiled by Dr. Cohen and consisted of 14 items and 2 dimensions [11]. PSS-14 was first applied to Chinese population in 2003 [12]. The Chinese version of PSS-14 scale adopted a 5-point scoring system (1 = never, 2 = almost never, 3 = sometimes, 4 = often, 5 = always). Of the 14 items, 8 items are scored in reverse. PSS score is obtained by adding the 14 items together, the higher the score the greater the perceived stress. The severity of the symptoms was divided into four grades: lower (14-28 scores) , moderate (29-42 scores) , higher (43-56 scores) and very high (57-70 scores) . The reliability and validity of this scale have been proved to be good, with a coefficient of 0.78 [12].

### Sampling method

A snowball sampling method was used in our study. The snowball sampling is a non-probability sampling method where new units are recruited by other units to form part of the sample. In our survey, a participant was asked to forward the same survey to his/her own WeChat circle of friends or other public platforms to expand the sample size after provided sociodemographic information and completed the questionnaire about stress sources. Each IP address could only be used once to avoid repeated question answering. The study was terminated two weeks after the increase in the number of filled questionnaires ceased.

We were aware of the endogeneity issue caused by a snowball sampling strategy. Therefore, we try to minimize such endogeneity and selection bias through orthogonal design of the questionnaire

### Ethical approval

The study protocol was reviewed and approved by Ethics Committee of the Affiliated Kangning Hospital of Wenzhou Medical University (YSSL2022008). The research was conducted in line with the Declaration of Helsinki and Good Clinical Practice. The aim and scope of the research were explained at the beginning of the survey in the questionnaire. A sentence on voluntary informed consent was added at the beginning of the questionnaire and participants that did not give voluntary informed consent were not allowed to continue the survey.

### Data analysis

Data were analyzed with IBM-SPSS Version 26.0 for window. Descriptive analysis was conducted to describe participants’ sociodemographic characteristics and situations causing stress (sources of stress). Normality of data was tested by the Kolmogorov-Smirnor test. Chi-square analysis was carried out to compare categorical data. Multivariate logistic regression analysis was performed to explore potential risk factors for perceived stress. Variables were included in the multivariate logistic regression analysis when they were statistically significant in Chi-square analysis. Adjusted odds ratio (OR) values and 95% confidence intervals (CI) were calculated using the multivariate logistic regression model. Statistical significance was set at α = 0.05, and all tests were two-tailed.

## Results

### Sociodemographic characteristics of participants

As shown in Table 1, a total of 488 participants participated in this online survey. The sociodemographic data of all the participants were categorized in terms of gender, age, employment status, education level, occupation, income, marital status, and location of residence. Moreover, the information related to COVID-19 epidemic was also included, including results of nucleic acid test, vaccinated or not, knowledge about COVID-19, and duration of home-quarantine. Some characteristics of the survey participants deserve to be highlighted: most (60.66%) of the participants were females; a considerable proportion (37.71%) of the participants were unemployed; majority (83%) of the participants were at a higher education level (undergraduate or postgraduate); a relatively high proportion (42.83%) of the participants were employed in medicine or education fields; a considerable proportion (29.51%) of the participants reported their income in the highest level ( ≥ 10,000 yuan/month); a vast majority (97.13%) of the participants were COVID-19 negative; only a small proportion (8.61%) of the participants were very knowledgeable about COVID-19; a vast majority (95.43%) of the participants had been vaccinated; a vast majority (95.90%) of the participants had been quarantined for ≥ 10 days.

### The stress sources perceived by home-quarantined residents

A total of 12 situations were considered major concerns and perceived stressful by the respondents. These situations were ranked from high to low based on the frequency they were mentioned by the respondents. As shown in Table 2, the top three concerns are “not allowed to go outdoors”, “uncertain duration of the epidemic”, and “lack of food supply”, each of these concerns was perceived stressful by more than 50% of the respondents. Fewer than 50% of the respondents perceived the other situations stressful, including “unsatisfied anti-pandemic measures” and “health of oneself and family members”.

**Table 2:** The stress sources perceived by home-quarantined residents (*N* = 488)

Sources of perceived stress	n	%
Not allowed to go outdoors	269	55.12
Uncertain duration of the pandemic	264	54.10
Lack of food supply	253	51.84
Lack of daily necessities	223	45.70
Unsatisfied anti-pandemic measures	221	45.29
Lack of physical activity	210	43.03
Health of oneself and family members	161	32.99
Job loss of oneself or family members	156	31.97
Income decrease	143	29.30
Traffic lockdown	121	24.80
Schooling for children	89	18.24
Unable to pay the loan	74	15.16

### The overall stress level and impact of sociodemographic factors

Of the 488 survey respondents, 207 persons (42.42%) were categorized into high stress level (PSS-14 score ≥ 43). Next, we analyzed the impact of each sociodemographic factor on the perceived stress of the respondents based on the proportion of people with high stress level. As shown in Table 3, some of them had significant impact on stress level, including age, employment status, gender, income (yuan/month), and marital status, whereas the others showed no significant impact, including location of residence, result of nucleic acid test, knowledge about COVID-19, whether vaccinated, and quarantine duration. Specifically, a highest proportion (52.49%) of the respondents in ≤ 29 years old group presented a high stress level (PSS-14 score ≥ 43) as compared to the other age groups. More male respondents (50.52%) showed a high stress level compared to female respondents (37.16%). More unemployed people (56.52%) reported a high stress level compared to the other groups. A higher proportion (49.18%) of singles perceived high stress compared to married people (35.65%). A highest proportion (54.78%) of people in the low income ( ≤ 1999 yuan/month) group showed high stress levels compared to the other groups.

**Table 3:** Impact of sociodemographic factors on perceived stress ( *N* = 488)

Sociodemographic factors	Subsamples N1	High stress (≥43 scores)			
		N2	% (N2/N1)	*Χ*^*2*^	*P*
**Employment status**				24.47	<0.01
Unemployment	184	104	56.52		
Employment	263	91	34.60		
Retired	41	12	29.27		
**Age (years)**				17.77	<0.01
≤29	221	116	52.49		
30-49	187	65	34.76		
≥50	80	25	31.25		
**Gender**				8.51	<0.01
Male	192	97	50.52		
Female	296	110	37.16		
**Marital status**				9.13	<0.01
Married	244	87	35.65		
Single	244	120	49.18		
**Income per month (RMB)**				16.08	<0.01
≤1999	157	86	54.78		
2000-4999	84	33	39.29		
5000-9999	103	41	39.81		
≥10000	144	47	32.64		
**Education level**				1.07	>0.05
High school or below	83	31	37.35		
Undergraduate	211	91	43.13		
Postgraduate	194	85	43.81		
**Occupation**				8.56	>0.05
Engineer or technique	52	24	46.15		
Business or IT	98	43	43.88		
Medicine or education	209	99	47.37		
Administer	26	9	34.62		
Agriculture or forestry	103	32	31.07		
**Location of residence**				1.96	>0.05
High risk area	243	97	39.92		
Medium risk area	142	67	47.18		
Low risk area	103	43	41.75		
**Nucleic acid test**				0.001	>0.05
Positive	14	6	42.86		
Negative	474	201	42.41		
**COVID-19 knowledge**				2.39	>0.05
No or little	274	110	40.15		
Some	172	75	43.60		
Very knowledgeable	42	22	52.38		
**Vaccinated**				0.25	>0.05
Yes	459	196	42.70		
Not yet	29	11	37.93		
**Quarantine (days)**				7.19	>0.05
≤9	20	12	0.60		
10-19	49	27	55.10		
20-29	201	77	38.31		
≥30	218	91	41.70		

### Risk factors for the perceived stress in the participants

The next goal of this study was to find out risk factors that increased stress level of the respondents who were quarantined at home during the early stage of COVID-19 epidemic. As shown in Table 4, males were more susceptible to COVID-19 and perceived high levels of stress relative to females (OR = 1.667, 95% CI = 1.140 – 2.437, *P* = 0.008). Unemployment is another risk factor of high stress as unemployed persons showed a higher OR of 2.822 (95% CI =1.346 – 5.917, *P* = 0.006) than the other groups. No other factors significantly increased the risk of stress perceived by the respondents. Although married people, people in early adulthood or in the income level of 5,000 – 9,999 yuan per month showed high values of OR relative to the other groups in their corresponding categories, the differences did not reach the pre-set significant level (*P* < 0.05).

**Table 4:** Protective or risk factors impacting the perceived stress

Variables	β	Wald χ^**2**^	***P***	***OR***	95% ***CI***
**Gender**					
Male	0.511	6.96	0.008	1.667	1.140-2.437
Female	0			1	
**Age**					
≤ 29	0.657	1.746	0.186	1.929	0.728-5.113
30~49	0.243	0.436	0.509	1.275	0.620-2.619
≥ 50	0			1	
**Work status**					
Unemployment	1.038	7.544	0.006	2.822	1.346-5.917
Employment	0.140	0.143	0.706	1.150	0.556-2.380
Retired	0			1	
**Income /month (Yuan)**					
≤1999	-0.02	0.002	0.967	0.98	0.378-2.541
2000~4999	0.054	0.016	0.899	1.056	0.456-2.443
5000~9999	0.486	2.24	0.135	1.626	0.860-3.074
≥10000	0			1	
**Marital status**					
Married	0.467	1.546	0.214	1.595	0.764-3.328
Single	0			1	

## Discussion

Home-quarantine is one of the most common measures applied to infected people with slight or without symptoms of SARS-CoV-2 to prevent or minimize the transmission of COVID-19 among communities [13–17]. However, the implementation of this measure is different in China, where all residents negative for COVID-19 in a medium or high risk area are required to stay at home while infected people must be hospitalized in the presence of clinical symptoms or collectively quarantined at a hotel or shelter hospital in the condition of no/slight symptoms before the end of 2022 [17, 18]. The home-quarantine implemented in the former case aims to minimize the transmission of COVID-19 from infected people to non-immune people, whereas the home-quarantine in the latter case intends to prevent non-infected people from contacting infected persons. This difference must lead to different consequences.

Obviously, China’s home-quarantine measure is more effective on minimizing or stopping the fast spread of COVID-19. Nevertheless, it more extensively impacts people’s mental health while restricting people’s daily life and damaging their family economic condition [17, 18]. Indeed, an online survey of more than 50,000 participants reported an incidence of 27.9% for depression, 31.6% for anxiety, nearly 30% for insomnia, and 24.4% for acute stress symptoms associated with COVID-19 in China [19]. In addition to infected persons and their families, frontline staff (medical staff and volunteers) and their families, close contacts of the infected persons, quarantined persons and those unemployed due to the epidemic are all vulnerable to mental and psychological problems related to the epidemic.

This online survey of a relatively small sample focused on impact of home-quarantine on mental health of residents lived in Shanghai during the early stage (from March to April 2022) of a new wave of COVID-19 pandemic. Overall, 42.42% of the survey respondents were at a high stress level. This proportion is considerably high relative to the data reported in previous studies. For example, a meta-analysis including 5 studies conducted before May 2020 with a total of 9074 participants showed an average of 29.6% heightened stress symptoms [20]. Similarly, an empirical research conducted from late April to mid-May 2020 showed 25% of moderate to extremely severe levels of stress [21]. The high level of perceived stress in survey respondents of this study may be related to the severity of the epidemic, residents' sense of fear and panic caused by COVID-19, and the strict control measures implemented by the government, such as restricting the flow of people, long home-quarantine, etc. Moreover, this high level of perceived stress does not necessarily mean that Chinese people are more vulnerable to COVID-19-related problems. This interpretation is in line with a recent study reporting that most residents showed normal levels of depression (71.3%), anxiety (67%), and stress (71%). About one-tenth reported mild levels of depression (11.6%), anxiety (8.4%), and stress (9.3%). The data of this report were collected over a period of 6 days, between 28 February 2020 and 5 March 2020, at the midpoint of the first wave of the COVID-19 outbreak in Hong Kong where most of residents are Chinese [22]. Therefore, we tend to claim that the restrictions applied to home-quarantined people during the epidemic of COVID-19 are important factors contributing to the high proportion of respondents with high level of stress in this study. They were not infected by COVID-19 thus were free from the direct effect of the infection.

In support of the above claim, most of the 12 situations considered by the respondents as stress sources are consequences of home-quarantine, while a few of them are indirectly related to COVID-19, including “uncertain duration of the epidemic”, “unsatisfied anti-pandemic measures”, and “health of oneself and family members.” The first six that were perceived stressful by relatively high proportion of respondents are “not allowed to go outdoors, 55.12%”, “uncertain duration of the epidemic, 54.10%”, “lack of food supply, 51.84%”, “lack of daily necessities, 45.70%”, unsatisfied anti-pandemic measures, 45.29%”, and “lack of physical activity, 43.03%”. Moreover, only a small part of the 12 sociodemographic factors showed significant impact on perceived stress of the respondents, including age, gender, marital status, employment status, and income, whereas the other factors had no effect, including education level, occupation, location of residence (low, medium, or high risk area), result of nucleic acid test, knowledge about COVID-19, vaccinated or not, and quarantine duration. Moreover, in our survey, the proportion of high stress of residents in different risk areas may be different, but the difference among them is not significant (χ^2^ = 1.96 *P* > 0.05). These results are different from our previous study, in which a higher proportion of people in high risk area showed severe anxiety (BAI score ≥ 30) compared to those in low risk area (9.49% vs 3.21%), and a much higher proportion of people at quarantine were categorized into severe anxiety compared to those not quarantined (66.67% vs 4.54%) [23].

The fact that COVID-19 related factors significantly increased anxiety level of the respondents in our previous study, whereas they showed no effect on stress level perceived by the participants in the present study is not surprising for the following evidence. The previous survey was done in February 2020, when the first wave of COVID-19 epidemic occurred, whereas the present study was conducted more than two years later. The first outbreak of COVID-19 did shock people even medical staff as the high fatality and pathogenicity of it. Over the past two and half years, however, advances in biological research, vaccination, preventive measures, and clinical treatment of COVID-19 never stopped, while the virus has kept mutating since the first identification of it in humans [10, 24, 25]. Although the new strains of the virus may be more transmissible, they are not necessarily more pathogenic as mentioned before. All these advances and changes would ameliorate the anxiety, stress, and panic of the public during the COVID-19 pandemic. But strict home-quarantine applied to people negative for COVID-19 would lead to a lot of mental health problems, including high levels of stress and anxiety.

Further support to the above conclusion, none of COVID-19 related factors is associated the stress level perceived by the respondents in this study, including location of residence (low, medium, or high risk area), nucleic acid test result, whether vaccinated, and quarantine duration. And very knowledgeable in COVID-19 and high education level showed no protective effect on the respondents in terms of stress level, whereas these are protective factors as shown in previous study [23]. However, males are more vulnerable (OR = 1.667, 95% CI = 1.14 - 2.44, *p* = 0.008) to home-quarantine than females, the unemployed were at higher risk of elevated stress (OR = 2.822, 95% CI = 1.35 – 5.92, *p* = 0.006) than those with work or retirement. Again, these results confirmed that the high level of stress perceived by the respondents in this study is more likely to be related to the long restrictions imposed to the home-quarantined people under the COVID-19.

In early December, 2022, soon after the submission of this manuscript, the China government put an end to the so-called “zero COVID” policy and abolished almost all restrictions that had been implemented before. A violent storm of COVID infection occurred across the whole country during a month and a half immediately following the revocation of the restrictions, then the epidemic almost stopped completely. Now people’s daily life is almost completely normal despite sporadic cases of COVID-19 infection which attract no attention anymore. These changes are in line with the conclusion from this study that home-quarantine applied to people negative for COVID-19 led to a lot of major concerns that may be perceived stressful.

### Limitations

We are aware of the limitations of this study. First, as the limited resources available and time-sensitivity of the COVID-19 epidemic, we used the snowball sampling strategy. This sampling method have a potential sampling bias and margin of error . This means a researcher might only be able to reach out to a small group of people and may not be able to complete the study with conclusive results. Second, although we corrected several covariates, some potential confounding effects cannot be excluded. The actual impact of independent variables used by the survey can be underestimated or overestimated. In addition, decisions based on inferences from the survey could be sub-optimal. This strategy was not based on a random selection of the sample, and the study population did not represent the actual pattern of the general population. Third, sample size was relatively small, thus did not allow further analysis on the data from some subgroups. Fourth, because the study is cross-sectional, it is unable to draw inferences regarding the cause-and-effect linkages between the variables.

## Conclusion

In conclusion, home-quarantine applied to people negative for COVID-19 led to a lot of major concerns that may be perceived stressful. In contrast, the virus-related factors did not show significant impact on mental health of the respondents. Therefore, it is time to reconsider the necessity of home-quarantine for COVID-19 negative populations, while continuing the measure for infected persons with no/mild symptoms.

## Data Availability

The datasets used and analyzed in this study are available from the corresponding author on reasonable request. Because of the sensitive nature of the data collected on the mental health of home-quarantined residents amongst which individuals are potentially identifiable, we cannot provide open access to our data.
